# Buffalo Academy of Medicine

**Published:** 1903-12

**Authors:** Wm. Irving Thornton


### Buffalo Academy of Medicine

*Section on Medicine, October 13, 1903.*

Reported by WM. IRVING THORNTON, M. D., Secretary.

The regular meeting of the medical section was held Tuesday evening, October 13, 1903, at the Academy rooms, in the Public

Library Building. The meeting was called to order at 8.45 o'clock by the chairman, Dr. ARTHUR W. HURD.

The minutes of the last meeting, held September 15, 1903, were read and approved.

Dr. DE LANCEY ROCHESTER presented a paper, entitled:

THE TREATMENT OF PNEUMONIA.

In opening he emphasised the fact that pneumonia was not to be considered a local disease of the lung only, but rather from its etiology, symptomatology and course a general systemic infectious fever. The first indication in treatment is the relief of toxemia. This is best brought about by means of catharsis, produced by salines or small doses of calomel, and by active diaphoresis. The latter indication is best met by the hot mustard foot bath, which is employed at intervals of 4 to 6 hours, and is continued from 30 to 45 minutes at a time, great care being taken that the patient is disturbed as little as possible and protected carefully from exposure. On account of the disease being so frequently complicated by nephritis, diuretics and added strain upon the kidney were considered inadvisable.

The diet is to be largely of liquids, either predigested or of easily digestible material. Large volumes of water are to be taken, but in small quantities at frequent intervals. In speaking of the local treatment of the lung conditions all applications of poultices and similar means were disapproved. The chest is to be protected by a woolen jacket. The frequent examination of the chest was urged and evidence of edema or spreading congestion to be met by the prompt application of leeches or by cupping. Applications of ice to the chest have been found unsatisfactory.

As soon as the diagnosis is made the condition is to be treated by moderate doses of strychnia, the dose to be increased as the disease progresses and the condition of the patient demands. The use of diffusible stimulants, such as alcohol, ammonium, carbonate and acetate and aromatic spirits of ammonia was commended.

To relieve the embarrassment of the right heart, bleeding alone or combined with hypodermoclysis of normal saline solution is useful. Digitalis carefully employed is of value in some cases., but its indiscriminate use was branded as irrational and unscientific. Some cases were reported treated with antipneumococcic serum, but he had not observed favorable results from its use.

Dr. Rochester reported nearly two hundred cases of pneumonia treated in private and hospital practice with most gratifying results, the mortality rate reported being but 5.55 per cent.

The paper was discussed by Drs. A. A. Jones, Grosvenor, Taylor, Schroter, Kennerson, Borzelleri, Fronczak, Stockton, B. G. Long, Wasdin and Van Peyma.

Dr. WILLIAM C. KRAUSS presented a short paper, entitled:

HYDROCEPHALUS—AN ATTEMPTED CLASSIFICATION.

He dealt briefly with the various varieties of the disease, speaking of the chief pathological states which produce the condition. He divided it into the following classes: acute—external, internal. Chronic—congenital, acquired.

Members present, 45. Adjourned at 10.30 p. m.

